# Characterization of the structure and interactions of P450 BM3 using hybrid mass spectrometry approaches

**DOI:** 10.1074/jbc.RA119.011630

**Published:** 2020-04-17

**Authors:** Laura N. Jeffreys, Kamila J. Pacholarz, Linus O. Johannissen, Hazel M. Girvan, Perdita E. Barran, Michael W. Voice, Andrew W. Munro

**Affiliations:** ‡ The Manchester Institute of Biotechnology, School of Natural Sciences, Department of Chemistry, The University of Manchester, 131 Princess Street, Manchester M1 7DN, United Kingdom; § Manchester Synthetic Biology Research Centre for Fine and Speciality Chemicals (SYNBIOCHEM), The University of Manchester, 131 Princess Street, Manchester M1 7DN, United Kingdom; ¶ Michael Barber Centre for Collaborative Mass Spectrometry, Manchester Institute of Biotechnology, The University of Manchester, 131 Princess Street, Manchester M1 7DN, United Kingdom; ‖ Cypex Ltd., 6 Tom McDonald Avenue, Dundee, DD2 1NH, United Kingdom

**Keywords:** cytochrome P450, dimerization, enzyme catalysis, enzyme structure, flavoprotein, fusion protein, mass spectrometry (MS), reductase, structural biology, collision induced unfolding (CIU), cytochrome P450 BM3, domain interactions, hydrogen-deuterium exchange mass spectrometry (HDX-MS), native ion mobility mass spectrometry (IM-MS), protein dynamics, protein solvent accessibility, monooxygenase, solution structure

## Abstract

The cytochrome P450 monooxygenase P450 BM3 (BM3) is a biotechnologically important and versatile enzyme capable of producing important compounds such as the medical drugs pravastatin and artemether, and the steroid hormone testosterone. BM3 is a natural fusion enzyme comprising two major domains: a cytochrome P450 (heme-binding) catalytic domain and a NADPH-cytochrome P450 reductase (CPR) domain containing FAD and FMN cofactors in distinct domains of the CPR. A crystal structure of full-length BM3 enzyme is not available in its monomeric or catalytically active dimeric state. In this study, we provide detailed insights into the protein-protein interactions that occur between domains in the BM3 enzyme and characterize molecular interactions within the BM3 dimer by using several hybrid mass spectrometry (MS) techniques, namely native ion mobility MS (IM-MS), collision-induced unfolding (CIU), and hydrogen-deuterium exchange MS (HDX-MS). These methods enable us to probe the structure, stoichiometry, and domain interactions in the ∼240 kDa BM3 dimeric complex. We obtained high-sequence coverage (88–99%) in the HDX-MS experiments for full-length BM3 and its component domains in both the ligand-free and ligand-bound states. We identified important protein interaction sites, in addition to sites corresponding to heme-CPR domain interactions at the dimeric interface. These findings bring us closer to understanding the structure and catalytic mechanism of P450 BM3.

## Introduction

Flavocytochrome P450 BM3 (BM3)^2^ is a natural fusion enzyme in which a fatty acid–binding cytochrome P450 (heme) domain (∼55 kDa) is fused to an NADPH-cytochrome P450 reductase (∼65 kDa) domain through a flexible interdomain linker region (1–3). BM3 is a biotechnologically important enzyme with engineered variants of BM3 having applications including drug metabolite production and the direct conversion of ethane to ethanol (4, 5). Genetic dissection of the full-length P450 BM3 enzyme has enabled the production of the individual heme and CPR domains, which retain properties characteristic of the full-length P450 BM3 enzyme (fatty acid binding and NADPH-binding/flavin reduction, respectively) (6–8). P450 BM3 was first isolated from the soil bacterium *Bacillus megaterium* and was found to hydroxylate a range of different saturated fatty acids with ∼12–18 carbon chain lengths at the ω-1 to ω-3 positions (9). The CPR domain binds NADPH and passes electrons from this cofactor through the CPR's FAD and FMN cofactors, and then from the FMN cofactor to the heme iron in the P450 domain of the enzyme (10). The consecutive transfer of two single electrons to the P450 heme iron enables the formation of first a ferric-superoxo species and then a ferric-peroxo species which undergoes two rapid protonation steps followed by a dehydration reaction to form the highly reactive compound I (Fe^IV^-oxo porphyrin radical cation) intermediate which catalyzes oxygen insertion into the substrate (11). The P450 BM3 structural arrangement allows for efficient electron transport from NADPH through the FAD, FMN, and heme cofactors, and results in P450 BM3 having the highest reported catalytic activity for a P450 monooxygenase enzyme (∼285 s^−1^ with arachidonic acid substrate) (12). In efforts to enhance activities of P450 enzymes, various groups have fused the reductase domain of BM3 to other P450s to produce catalytically self-sufficient flavocytochromes with improved catalytic rates (13). Other homologues of P450 BM3 have also been characterized (14).

**Figure 1.**
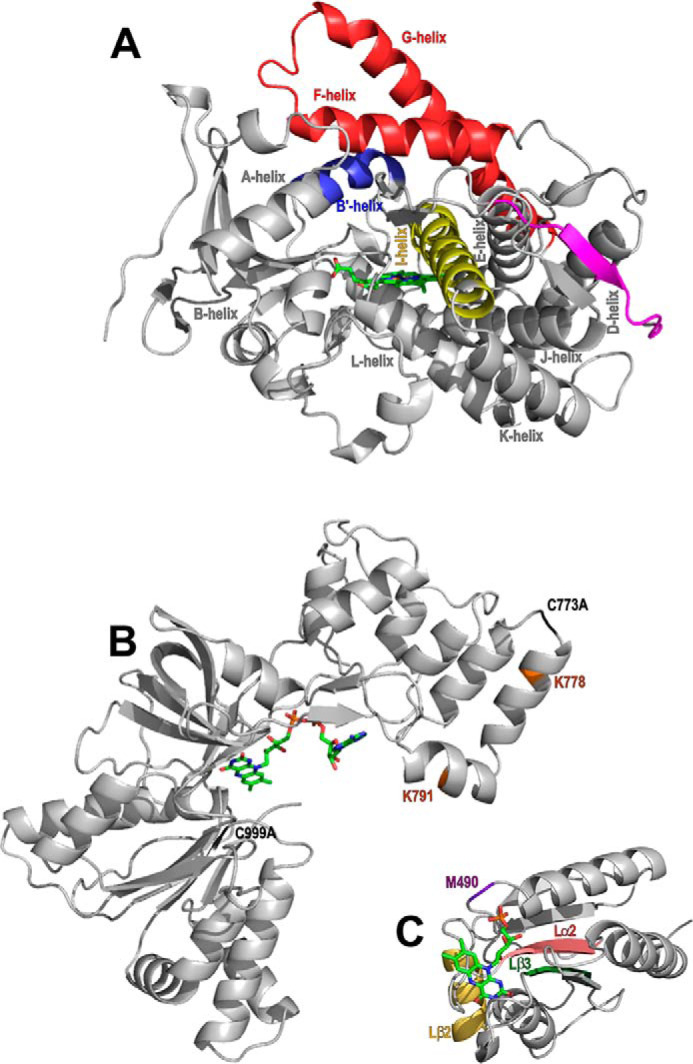
**The structure and important features of P450 BM3 and its domains.** The P450 BM3 heme domain is the catalytic domain and is presented as the 1BU7 structure (16). The CPR domain contains an FMN-binding domain and an FAD/NADPH-binding domain. The structures of these individual subdomains have been determined and are shown in the structures 1BVY (16) and 4DQK (15), respectively. *A*, the 55-kDa heme domain of P450 BM3 contains a heme prosthetic group (*green*) and is responsible for substrate binding and oxidative catalysis of the substrate. The I-helix (*yellow*) and the F/G-helices (*red*) are highly flexible components of the P450 fold. Other regions of the P450 fold shown are the mobile B′-helix (*blue*) and the heme domain C terminus (*magenta*) close to the heme prosthetic group (*green*). Other selected P450 α helices have also been labeled. *B* and *C*, the 65 kDa CPR domain obtains electrons from NADPH for the reductase activity of the enzyme. The CPR contains two subdomains; the FAD/NADPH-binding domain (*B*) and the FMN-binding domain (*C*). *B*, the two amino acid mutations required for inhibition of FAD domain dimer formation (C773A and C999A) that are necessary for successful FAD domain crystallization are highlighted in *light green* (15). In *light blue*, two lysine residues (Lys-778 and Lys-791) are colored *brown* and are found close to the CPR dimeric interface, as shown by EM experiments. The inter-CPR distances are ∼20 Å between Lys-778/Lys-791 in both cases (21). *C*, regions of the FMN-binding (flavodoxin) domain found to be important from dynamic simulations for electron transfer to the heme prosthetic group are highlighted in *purple*, *wheat*, *dark green*, and *pink* (32).

Although several crystal structures have been determined for WT and mutant forms of the BM3 heme domain, no structures have been solved for the intact CPR domain, or for the full-length (monomeric or dimeric) P450 BM3 protein. However, structures are available for the FAD/NADPH-binding (ferredoxin reductase-like) FAD domain of the BM3 CPR (15), and the structure of the FMN-binding (flavodoxin-like) FMN domain has also been solved as part of a P450 BM3 structure from which the terminal FAD/NADPH-binding domain was removed, as shown in Fig. 1. In this truncated heme-FMN domain structure, the FMN domain was cleaved from its heme domain during purification or crystallography but could still be resolved as part of a structure which contained two heme domains and one FMN domain in each asymmetric unit (16). This is currently the only known crystal structure of the P450 BM3 FMN-domain. Full-length P450 BM3 is a functional dimer, with the dimeric interface present in the CPR domain (17). In the FAD domain structure, two surface cysteine residues were mutated to prevent adventitious disulfide bridge formation that could otherwise lead to a dimeric state of the FAD domain. This strategy enabled the successful crystallization of the monomeric form of this domain (15).

Aside from X-ray crystallographic approaches, several other methods have been used to characterize the structural and spectroscopic properties of P450 BM3 and its component domains. These include techniques such as CD (for secondary structural analysis), Mössbauer spectroscopy (to probe ferryl species in BM3 and P450cam), resonance Raman (for analysis of heme structure and heme iron coordination), and EPR (for characterization of heme radical species) (18–21). However, the full-length P450 BM3 and its component heme and CPR domains are not readily amenable to structural analysis by NMR approaches because of the large sizes of these proteins and the paramagnetic nature of the ferric iron in the heme prosthetic group.

Recently, a full-length structure of a P450 BM3 dimer was modeled using data collected from negative stain and 2D electron microscopy (EM) studies. In the EM structural model, the two CPR domains interact with one another in an extended conformation, with the more flexible N-terminal heme domains predicted to interact with the CPR FMN domains at the proximal face of the heme. In this model, the CPR domains are tightly associated whereas the heme domains can occupy multiple conformations. However, the exact positions of the heme domains could not be accurately determined and so specific residues or regions of interaction with the CPR FAD-/FMN–binding domains could not be defined (22). These data are in contrast to a solution state small angle X-ray scattering study, in which the human CPR enzyme was shown to exist in two conformations that could be described as “open” and “closed” states with respect to the packing of the FMN domain against the FAD domain, and in which the FMN domain was described as a ball on a string (23). This model is supported by other studies on the P450 BM3 enzyme which suggest that the FMN-binding domain shuttles electrons to the P450 heme iron by a FMN domain movement toward the heme domain, following its obtaining electrons from the FAD domain. In studies by Neeli *et al.* (17) and Girvan *et al.* (24), electron transfer was shown to occur within a P450 BM3 dimer and between the FMN domain of one of the monomers and the heme domain of the other. This model is consistent with the properties of the related flavocytochrome nitric oxide synthase (25). In contrast, Kitazume *et al.* (26) suggested a model in which the FAD domain of one monomer reduces the FMN domain of the other monomer within the CPR. Thus, there are still several questions to be answered regarding the catalytic mechanism and molecular dynamics of the biotechnologically important P450 BM3 enzyme.

In previous studies, we have used a variety of MS techniques including hydrogen-deuterium exchange mass spectrometry (HDX-MS), native MS, and CIU to analyze structural properties and dynamics within proteins that have been difficult to capture by alternative methods, such as X-ray crystallography (27). The HDX-MS technique involves diluting the target protein in deuterated buffer for different lengths of time to allow for the exchange between backbone (amide) hydrogens and deuterium from the solvent. The protein is then digested on a protease column and the resulting peptides are analyzed. The Pikuleva lab published the first HDX-MS data for a P450 enzyme and used these data to identify an allosteric binding site on CYP46A1 (28, 29). Since then HDX has been used to elucidate information about the structures of many other P450 enzymes such as CYP2B4, CYP3A4, and P450_cam_ (30–32)_._ In this paper, we have used HDX-MS to map the protein surface of P450 BM3 to gain insights into the dimeric interface(s) of the enzyme.

## Results

### Native MS

Native MS experiments confirmed that the full-length protein exists predominantly as a dimer in solution (Fig. S1*A*). The monomeric heme domain exhibited the same three charged species in its ligand-free and NPG-bound states, with the 14+ state being the most prominent (Fig. S1, *B* and *C*). For the CPR (diflavin reductase) domain, multiple species were observed, corresponding to one, two, three, and four molecules of NADP^+^ bound to the reductase domain (Fig. S1, *D* and *E*).

### Collision-induced unfolding and ion mobility-MS

**Figure 2.**
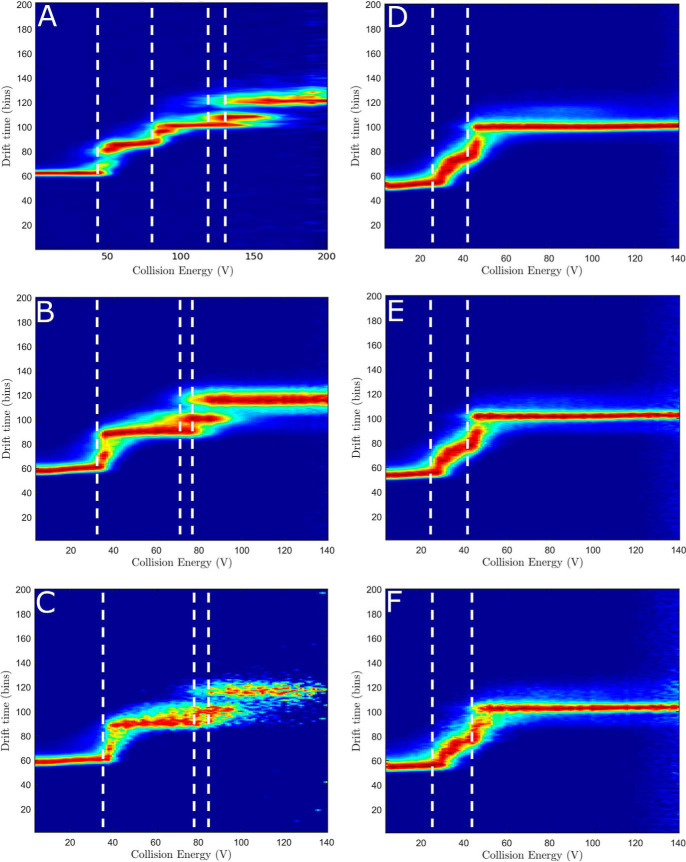
**Collision-induced unfolding of the full-length P450 BM3 enzyme and its P450 and reductase domains.** CIU was undertaken on the full-length P450 BM3 using the most prevalent charge states found in the native MS studies, shown in Fig. S1. *A*, the ligand-free, full-length P450 BM3 using charge state [33+] shows six unfolding events. *B*, the ligand-free BM3 heme domain using charge state [14+] shows three unfolding events. *C*, the NPG-bound BM3 heme domain using charge state [14+] shows an increase in stability of the protein, as slightly more energy is required before unfolding is initiated. *D*, the BM3 reductase (CPR) domain using charge state [16+] shows a range of overlapping intermediates. *E* and *F*, the BM3 CPR domain bound to NADP^+^ using charge state [16+] shows no change to the unfolding pattern that was observed with the ligand-free CPR domain. The stoichiometry used was 1:1 (*E*) and 1:2 (*F*) protein to NADP^+^.

Collision-induced unfolding (CIU) is a technique where external energy is applied to probe protein stability and follows protein unfolding, similar to methods in which increasing temperature is used to unfold the protein, such as differential scanning calorimetry (DSC) (4). The unfolding intermediates are also visualized using this method. CIU was performed on ligand-free domains or full-length dimeric protein (Fig. 2, *A*, *B*, and *D*) and ligand-bound domains (Fig. 2, *C*, *E*, *F*). The ligand-free, full-length dimeric P450 BM3 protein showed six discrete unfolding events (Fig. 2*A*). The heme domain showed three discrete unfolding intermediates (Fig. 2*B*), whereas the CPR domain undergoes unfolding across a narrow range of overlapping, unfolding intermediates (Fig. 2*D*). As stated previously, the CPR module contains two domains: the FAD domain and the FMN domain. However, the CPR domain exhibits an unfolding pattern more suggestive of a single domain structure. In contrast, the heme domain behaves more like a multidomain structure during CIU experiments, because of the presence of three discrete unfolding events. The full-length dimeric protein and its domains remain stable in their unfolded states up to 200 V as there is no loss of signal.

The collision cross section (^TW^CCS_He_) values derived from IM-MS were 34.6 ± 0.2, 37.5 ± 1.1, and 99.7 ± 1.2 nm^2^ for the heme domain, CPR domain, and full-length dimeric BM3 protein, respectively (shown in Table S1). A CPR model structure was produced using the FMN-binding domain structure (PDB 1BVY) and the FAD-binding domain (PDB 4DQK) aligned to the NADP^+^-bound rat CPR (PDB 1AMO). This model was used throughout the work described here and will be referred to as the CPR model structure from this point forward. These structures gave CCS values of 39.1 nm^2^ for the heme domain (13% difference to the experimental data) and 46.1 nm^2^ for the CPR domain model (23% difference to the experimental data). Highly dynamic and flexible proteins were shown to have significantly smaller experimental CCS when compared with theoretical CCS based on crystal structures, because of compaction in the gas phase (33).

The binding of NPG to the heme domain slightly increased the unfolding energy barrier of the heme domain (Fig. 2*C*). The undefined edges are because of the ligand dissociating at high voltages. The presence of NADP^+^ did not appear to have any significant effect on the unfolding energy barrier of the CPR domain (Fig. 2, *E* and *F*).

### Hydrogen-deuterium exchange MS

**Figure 3.**
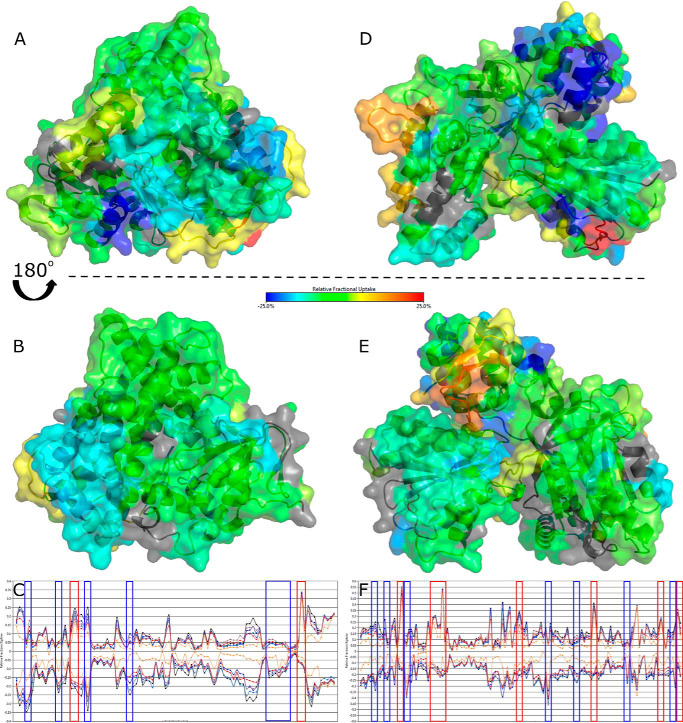
**HDX-MS of the surface of the P450 BM3 heme and CPR domains compared with the full-length dimeric protein.** Comparisons of the heme and CPR domains to the full-length dimeric P450 BM3 show areas of increased shielding (*cyan* and *dark blue*) and solvent accessibility because of deshielding (*orange* and *red*). Residues with no coverage are displayed in *gray*. The crystal structures used are 1BU7 (heme domain) and 1BVY/4DQK (FMN-binding domain and FAD/NADPH-binding domain, respectively) aligned to the rat CPR 1AMO structure as a CPR model. Residues with significant deuterium uptake changes are shown in Fig. S2. *A* and *B*, comparing the heme domain to the heme domain of the full-length dimeric P450 BM3 protein shows some areas of change. The area with the greatest shielding (*dark blue*) when the reductase domain is present is from residues 22–30 on the distal face of the protein, seen clearly in *A*. The area of greatest solvent accessibility centers around glutamic acid 424 (*red*). Coverage maps for the HDX-MS heme domain data are shown in Fig. S3*A*. *D* and *E*, the CPR domain exhibits a highly dynamic structure, as shown by large changes in deuterium uptake. Many areas within the full-length P450 BM3 protein are deshielded, leading to increasing deuterium exchange (*orange-red*). In particular, a portion of the hinge domain of the FAD-binding domain (residues 721–729 in *yellow* and residues 732–748 in *orange*) and the corresponding shielded areas (residues 709–720, 766–767, and 773–781 in *dark blue*) suggest significant protein movement. Coverage maps for the HDX-MS CPR domain data are shown in Fig. S3*B*. *C–F*, butterfly plots show the difference in relative deuterium uptake for each peptide fragment for the heme and CPR domains compared with the full-length protein, respectively. The upper states in both plots correspond to the full-length protein and the lower states to the heme (*C*) and CPR (*F*) domains. Multiple time points for deuterium uptake are observed for each peptide: 1 min (*orange*), 10 min (*red*), 30 min (*gray*), 60 min (*light blue*), 180 min (*dark blue*), and 480 min (*black*). *Vertical lines* enclose regions of greatest change, as exhibited in *A*, *B*, *D*, and *E*, with *blue* corresponding to shielding and *red* corresponding to deshielding for the full-length P450 BM3 dimeric protein compared with its domains.

HDX-MS is a technique that enables visualization of the solvent accessibility of amino acid residues to determine protein structure, ligand binding, and protein dynamics. Here we have performed experiments comparing two states to each other, for example by contrasting a full-length protein with its component domains, or by comparing a ligand-free protein with a ligand-bound protein to determine which areas become more shielded (less deuterium uptake) or more solvent accessible/deshielded (more deuterium uptake) on conformational rearrangement. Some examples of deuterium plots showing the changes in deuterium uptake over time across several states are shown in Fig. S2. Fig. 3 compares the heme and CPR domains of P450 BM3 to the dimeric full-length BM3, showing areas of increased shielding (*green* to *blue*) and solvent accessibility because of deshielding (*yellow* to *red*). Fig. 3, *A* and *B*, show the differences between the heme domain of the full-length P450 BM3 and the heme domain mapped onto PDB 1BU7 and rotated by ∼180 degrees. Differences were calculated by subtracting the heme domain deuterium values from the full-length P450 BM3 deuterium values. A large area of Fig. 3*B* in the heme domain from the full-length P450 BM3 shows a slight increase in shielding (*cyan*), including residues 279–315 (encompassing the I-, J-, and J′-helices and the β5 sheet). Other areas that see changes include the F-helix (residues 178–185) and the β3/4 sheets in *cyan*. The area with the greatest shielding (*dark blue*) when the CPR domain is present is from residues 22–30 in the heme domain A-helix, as seen clearly in Fig. 3*A*. These areas are potential sites for forming the interface between the CPR and heme domains. Interestingly, there are certain areas (shown in Fig. 3*A*) that have increased solvent accessibility when the CPR domain is present in the protein, in particular, glutamate 424 (*red*) and the residues surrounding it (*yellow*). Fig. 3*C* shows the butterfly difference plot for the HDX-MS data, highlighting areas of greatest change for the heme domain in *blue* (indicating increased shielding) and *red* (indicating increased deshielding). Similarly, the greatest changes observed for the CPR domain, by comparing this domain to the full-length protein, are highlighted in Fig. 3*F*.

Fig. 3, *D* and *E* shows that the isolated CPR domain exhibits greater changes in the uptake of deuterium than for full-length P450 BM3 or the heme domain, as observed for the *blue* and *red* regions of the protein. Differences were calculated by subtracting the CPR domain deuterium values from the full-length P450 BM3 deuterium values. These data have been mapped onto the CPR model structure (PDB 1BVY/4DQK aligned to PDB 1AMO). Many areas within the full-length P450 BM3 protein are deshielded, leading to increased deuterium exchange (*orange* to *red*). In particular, a portion of the hinge domain (linking the FAD and FMN domains, including residues 721–729 in *yellow* and residues 732–748 in *orange*), a region adjacent to the NADPH-binding site of the FAD domain (residues 967–974 in *orange* and residues 980–990 in *yellow*) and a portion of the FMN domain (residues 593–598 in *red*). These data show how flexible the CPR domain is and how much it moves in the presence of the heme domain within the full-length P450 BM3 protein. Examination of the shielded areas for the interaction sites of the two domains reveals a number of interesting regions, indicated in *dark blue* in Fig. 3, *D* and *E*. In the FAD domain, three areas are identified in the hinge domain: residues 709–720, 766–767, and 773–781 in *dark blue*. In the FMN domain, the residues most affected are 559–566 (*cyan*), Thr-592 (*dark blue*) and 599–601 (*dark blue*). The combination of shielded and deshielded areas in the CPR hinge domain suggests a dramatic protein movement, allowing one side to become more solvent accessible and the other more shielded.

**Figure 4.**
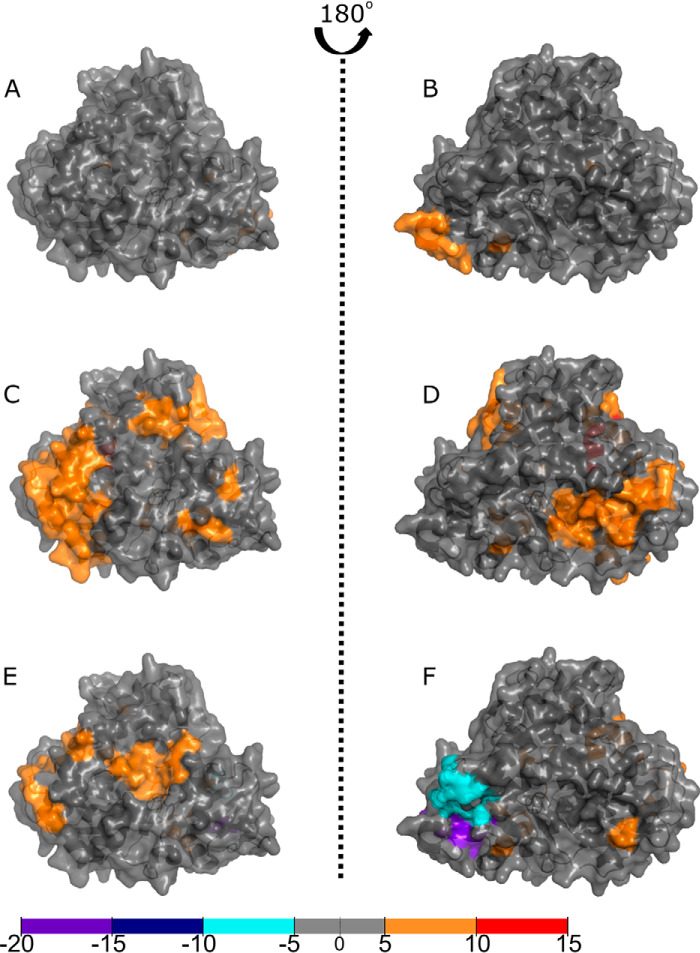
**Binding of ligands to full-length P450 BM3 elicits structural rearrangements across the heme domain as visualized by HDX-MS.** Comparisons of the P450 BM3 heme domain with the full-length P450 BM3 enzyme in substrate-free or ligand-bound form reveal areas of increased shielding (*cyan*, *dark blue*, and *purple*) and solvent accessibility because of deshielding (*orange* and *red*). The crystal structure used is that of the NPG-bound heme domain (1JPZ). Residues with significant deuterium uptake changes are shown in Fig. S2. *A* and *B*, comparisons of the ligand-free heme domain with that of the NADP^+^-bound protein reveal very little change in the heme domain structure. *C* and *D*, comparisons of the ligand-free heme domain with the NPG-bound form of the protein show that the majority of the protein has undergone deshielding, as revealed by the widespread *orange* coloring. *E* and *F*, comparisons of the ligand-free heme domain to the NADP^+^/NPG-bound form of the protein show slight differences across the ligand-free heme domain compared with the NPG-bound form (*C* and *D*). Coverage maps for the HDX-MS heme domain data are shown in Fig. S4.

**Figure 5.**
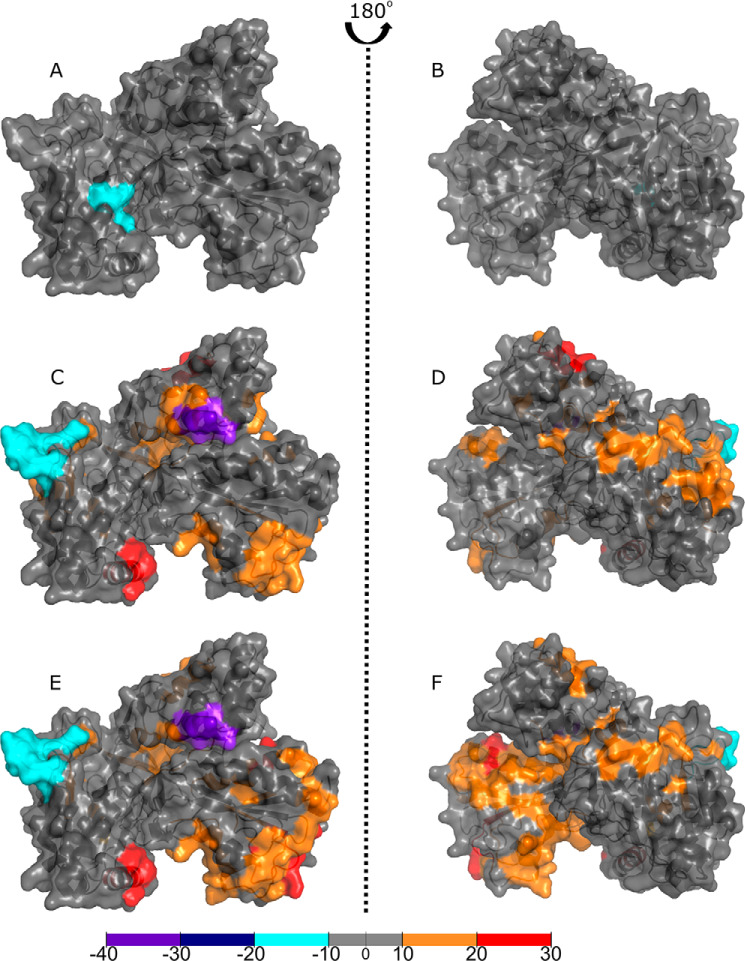
**Binding of ligands to full-length P450 BM3 elicits structural rearrangements across the CPR domain as visualized by HDX-MS.** Comparisons of the CPR domain with the full-length P450 BM3 protein in the substrate-free or ligand-bound forms reveal areas of increased shielding (*cyan*, *dark blue*, and *purple*) and solvent accessibility because of deshielding (*orange* and *red*). The crystal structure used is the rat CPR model produced by aligning the BM3 FMN domain from the 1BVY structure and the BM3 FAD domain from the 4DQK structure to the rat CPR structure (1AMO). Residues with significant deuterium uptake changes are shown in Fig. S2. *A* and *B*, comparisons of the ligand-free CPR to the NADP^+^-bound form of the protein revealed little difference in solvent accessibility, except for one area of deshielding within the FAD domain. *C* and *D*, comparisons of the ligand-free CPR with the NPG-bound form of the full-length P450 BM3 protein showed that much of the CPR domain has undergone a change toward *orange* and *red*, indicating greater solvent accessibility that is likely because of the CPR domain adopting a less compact conformation. The greatest decrease in deuterium uptake is seen on the hinge domain of the FAD domain. In comparison, the greatest increase in deuterium uptake for the FAD-binding domain is located on the opposite face of the hinge domain (*purple*). *E* and *F*, binding of NPG elicits the greatest change across the CPR domain, whether NPG only or both NADP^+^/NPG were bound, as observed in *C–F*. There is a greater shift from *gray* to *orange*, indicating a larger conformational reorganization. The areas with the greatest shielding observed are present in the NPG- and NADP^+^/NPG-bound proteins. Coverage maps for the HDX-MS CPR domain data are shown in Figs. S3B, S5 and S6 describe, respectively, (i) P450 BM3 residues that exhibit highest degrees of change when full-length P450 BM3 is compared with its isolated heme and CPR domains; and (ii) P450 BM3 residues that exhibit highest degrees of change when comparing ligand-free and ligand-bound forms of P450 BM3.

Figs. 4 and 5 compare the effects of ligand binding to the full-length P450 BM3 protein. Differences were calculated by subtracting the ligand-free, full-length P450 BM3 deuterium values from the ligand-bound full-length P450 BM3 deuterium values. As no full-length structure exists presently, the data have been mapped onto the ligand-bound heme domain crystal structure PDB 1JPZ and the CPR model used previously (PDB 1BVY/4DQK aligned to PDB 1AMO). The ligands used for these experiments were the substrate NPG (which binds within the P450 active site) and NADP^+^, which binds adjacent to the FAD cofactor of the CPR domain). The NADP^+^-binding site is immediately adjacent to the FAD isoalloxazine ring, but the oxidized NADP^+^ cannot reduce the FAD and instead acts as a structural mimic of NADPH. In Fig. 4, *A* and *B*, the ligand-free protein is compared with the NADP^+^-bound protein, revealing very little structural change in the heme domain. There is a slight increase in solvent accessibility visible around the N terminus of the heme domain. In particular, three residues of the A-helix, near the N terminus, exhibit deshielding in all ligand-bound states. The greatest change to the heme domain was exhibited on NPG binding or NADP^+^/NPG binding to the full-length P450 BM3 in Fig. 4, *C* and *D*, and *E* and *F*, respectively. Both states exhibit deshielding around residue 138 and a portion of the J-helix (residues 294–305). However, these areas are more extensive for the NPG-bound protein. No significant shielding is observed for the NPG-bound protein. However, deshielding is observed across the protein upon NPG-binding in the B-, B′-, C-, C′-, D-, E-, F-, I-, J-, J′-, and K-helices. In addition, a β sheet at the C terminus of the heme domain exhibits significant deshielding. However, the greatest deshielding is observed in the E-helix (residues 149–155 in *red*). In contrast, the NADP^+^/NPG-bound protein exhibits fewer deshielded areas, many identical to the NPG-bound protein. This differs for the large deshielded area containing much of the L-helix, observed on NADP^+^/NPG-binding. Interestingly, shielding is observed for the NADP^+^/NPG-bound protein near the N terminus (residues 15–20 in *purple*) and the helices and random coil segments between the F- and G-helices (residues 187–197 in *cyan*).

In Fig. 5, the CPR domain exhibits greater surface changes than does the heme domain on ligand binding. Comparing the ligand-free CPR domain to the NADP^+^-bound form reveals very little change in solvent accessibility across the CPR domain, shown in *gray* (Fig. 5, *A* and *B*). There is also a slight increase in shielding in the FAD domain for residues 1007–1011 (*cyan*). Surprisingly, binding of NPG had a greater effect on the conformational changes exhibited in the CPR domain compared with those induced by the binding of the CPR domain ligand NADP^+^, as seen in Fig. 5, *C* and *D* (NPG-bound) and *E* and *F* (NADP^+^/NPG-bound). The figures illustrate that the CPR domain has undergone many structural changes, including extensive deshielding of the FMN domain, which is further amplified in the NADP^+^/NPG-bound structure. This is shown, in particular, by *orange* segments observed in the FMN domain (residues 559–567) and in the FAD domain (residues 711–720 and 822–829). In contrast, shielding is observed for the FAD domain in two areas: residues 967–974 (in *cyan*) and residues 787–792. The latter area is located on the opposite face to the greatest deshielded area of the protein (residues 713–718). For the NADP^+^/NPG-bound CPR structure, there is an additional increase in solvent accessibility in two areas: residues 1029–1034 of the FAD domain (*red*) and residues 559–564 of the FMN domain.

## Discussion

Comparison of the experimental CCS values to the CCS values calculated from the crystal structures showed slight differences for the heme and CPR domains. Because of the lack of a full-length crystal structure of P450 BM3, no CCS values could be predicted in this case. The isolated heme domain CCS value discrepancy is within the 14% range described as an acceptable error in the literature (34). However, the CCS value for the CPR domain is outside this error range, suggesting that our model may not be a highly accurate representation of the CPR in solution. Our experimental data suggest that the P450 BM3 CPR domain adopts a more compact conformation than rat CPR when in its ligand-free form.

From thermal denaturation (DSC) experiments, the full-length P450 BM3 was found to have three distinct unfolding events, with the isolated heme domain having two unfolding events and the CPR domain a single unfolding event (4, 17). Our CIU data suggest additional unfolding events for the full-length P450 BM3 and the heme domain. For full-length P450 BM3, three unfolding events are very clearly defined with prolonged drift time over a large range of collision energies. There are also three much shorter, overlapping unfolding events which might make these events difficult to identify using techniques such as DSC. A similar overlapping series of unfolding events is observed in the heme domain CIU experiments. Other experiments also confirmed the stabilization of the heme domain upon substrate binding, with the tight-binding NPG increasing the major heme domain melting temperature by ∼4.7 °C (4). Our CIU experiments show little difference between ligand-free and NPG-bound heme domain protein, except for a loss in the quality of signal because of poor ionization.

Comparisons between the full-length P450 BM3 protein and the heme domain during HDX-MS studies reveal many areas of shielding, including those on the proximal surface of the protein where the redox partner (CPR) domain interacts to catalyze electron transfer to the P450 heme iron. Gricman *et al.* (35) and others have studied P450-redox partner interactions in P450 BM3 using the truncated BM3 heme-FMN domain crystal structure (1BVY) as this is currently the only crystal structure of the FMN-domain available for modeling. However, this structure is formed with a nonstoichiometric ratio of two heme domains to one FMN domain, with the single FMN domain cleaved from its heme domain and positioned distant from the heme prosthetic groups. Computational analysis indicated that electron transfer from FMN to heme in the conformation observed in the crystal structure would take ∼50 years to complete for an extended ∼50-σ bond route (through amino acids and their peptide bonds) from FMN to heme. This is unlikely to be an efficient electron tunneling pathway, and an alternative route of ∼18 Å between conjugated edges of the FMN cofactor and the heme prosthetic group was calculated to have an electron transfer rate constant of ∼12 s^−1^ (1, 16, 35). Despite this, modeling of the interactions between the BM3 heme domain and the FMN domain identifies larger regions of interest from the 1BVY structure. In particular, molecular modeling reveals a number of residues and regions that are highly mobile, resulting in three collective movements that lead to a more compact conformation of the heme-FMN complex that would be required for a high catalytic rate of FMN to heme iron electron transfer to be achieved (36).

Comparing the identified residues with our HDX-MS approach shows that they align to the highly dynamic areas in the heme domain, as observed by comparing the heme domain to the full-length P450 BM3, shaded *red* and *cyan* in Fig. 3 (36). Darimont *et al.* (37) mutated these residues (found within the K- and L-helices, as well as random coil segments of the heme domain and M490 of the FMN-binding domain) and found that electron transfer coupling efficiency and enzyme activity could be greatly altered by engineering these residues, but only when the redox reaction was driven by NADPH (the physiological cofactor), and not when an electrode system was used. Our data reveal areas that are not explained by these collective movements, but which exhibit some of the greatest changes in deuterium uptake. This suggests that other factors may contribute, such as shielding and deshielding by structural changes in the dimeric interface. These areas include residues 22–30, which have the greatest shielding observed across the heme domain surface, and a large amino acid stretch from residues 294–305 (within the I- and J-helices). Through modeling the surface charge using PyMOL, the segment containing amino acids 22–30 was shown to have a modest positive charge, whereas the larger segment from amino acids 294–305 exhibits a small negative charge. The shielded regions are adjacent to each other and close to other deshielded areas, suggesting a movement in this area, when comparing full-length P450 BM3 with the heme domain protein, which may be attributed to the conformational changes occurring during dimerization as the isolated heme domain is not capable of dimerization.

For the full-length P450 BM3, the heme domain did not exhibit structural changes as dramatic as those observed for the CPR domain on ligand binding. Comparisons of the data described here to those for the human cholesterol 24-hydroxylase CYP46A1 (the only other published example of P450 HDX-MS data) revealed similar percentage changes in deuterium uptake (28, 29). Areas of shielding were formed near the N terminus on NADP^+^/NPG binding, in the same region as observed when comparing the full-length dimeric protein to the heme domain. In contrast, the other area of shielding (in the J-helix) showed a slight deshielding on NPG binding. Also of interest is the consistent deshielding of Thr-149 in the E-helix observed on NADP^+^ binding. This residue is not well-represented in the literature and no mutagenesis studies have targeted it to date.

Unfortunately, there are no crystal structures for the full-length P450 BM3 enzyme in which the catalytically important heme-FMN linker region is intact, and thus we could not map our HDX data accurately to this critically important, but highly dynamic, region of P450 BM3. The deuterium uptake changes observed for this linker region were relatively low; between 4.8 to 6.1% by comparing the CPR and heme domains to the full-length P450 BM3 protein, and between −1.5 to 2.7% for the NADP^+^/NPG-bound forms compared with the ligand-free full-length P450 BM3. Examination of the raw data without state comparisons indicated that the linker region had very high deuterium uptake (8–27%) whether in its full-length form (ligand-free or ligand-bound) or in the CPR domain (ligand-free), which is unsurprising as the flexible linker region likely has very high solvent accessibility regardless of the protein conformation.

We were able to map the majority of the CPR domain data using the CPR model (PDB 1BVY/4DQK aligned to PDB 1AMO), revealing a number of shielded areas throughout this domain. However, our focus was drawn toward the FMN domain, as this protein module is known to be responsible for shuttling NADPH-derived electrons between the BM3 CPR and heme domains (17, 24–26). The highly dynamic regions of the FMN domain determined by modeling 1BVY were found to be Lβ2/3 and Lα2 (36). Comparisons between the CPR domain and the full-length P450 BM3 revealed that these highly dynamic β sheets exhibited profound shielding, whereas the α helix had slightly increased deshielding. In particular, there is a substantially shielded area adjacent to a highly solvent accessible area of the FMN domain from residues 592–602. This corresponds to a very negatively charged region of the protein.

Within the CPR domain, lysine residues 778 and 791, previously described as key residues involved in forming the dimeric interface from the EM structure, sit either side of the most shielded area in the hinge region of the FAD domain when comparing full-length P450 BM3 to the CPR domain (22). When comparing NPG-bound to ligand-free data for the CPR domain of the full-length protein, the area of greatest shielding within the hinge domain becomes slightly shifted in location and is focused around lysine 791. Interestingly, the other side of this hinge domain has greatly increased solvent accessibility, indicating substantial protein movement in this area. The cysteine residues that affect the crystallization of the FAD-binding domain display decreased deuterium uptake corresponding to a highly shielded area when comparing full-length P450 BM3 to the CPR domain (Fig. 3, *D* and *E*). A C773A mutation was originally introduced to prevent dimerization of the FAD-binding domain (through formation of an intermolecular disulfide bridge) and to facilitate its crystallization, and is located in the CPR hinge domain region (15). Coverage for this residue was missing for the ligand-bound forms. However, where coverage was available near this residue, shielding was observed. The areas of greatest deuterium uptake observed in the CPR domain, particularly from the full-length P450 BM3 to CPR domain comparison, are also those regions that were suggested for interactions in the small angle X-ray scattering models for the human CPR (23). The CPR domain is able to dimerize in solution, suggesting that the shielding we observe in this hinge domain is also consistent with other data in the literature, and is associated with the site of the CPR domain dimerization. Consolidating our domain data with our ligand-bound data suggests that the CPR dimerization interface undergoes a slight rotation on ligand-binding, causing the shielding of residues 787–792 and the deshielding of residues 711–720.

NADP^+^ binding had a small effect on the structure of the BM3 CPR domain, with various shielding and deshielding events observed across the protein surface. The largest changes observed occur in the hinge domain. NPG binding elicits substantial changes across the CPR domain in full-length P450 BM3, which are further amplified in the NADP^+^/NPG-bound structure. This suggests that binding of a substrate (NPG) to the heme domain induces major conformational changes that are transmitted through to the CPR domain, and that the binding of NADP^+^ produces other conformational changes that are conducive for catalysis. The areas of greatest deshielding are seen in (i) the hinge domain of the CPR, (ii) within the β sheets of the CPR FMN domain, and (iii) in a peripheral α helix within the FMN domain (Lα2). Studies have suggested that NADP^+^ binding to the CPR domain induces a closed conformation in which the FAD and FMN cofactors are a short distance apart (∼4 Å) (38). On reduction by NADPH, an open conformation is adopted as the FAD/NADPH- and FMN-binding domains move apart to enable FMN domain/heme domain interactions (38). An important residue for the dissociation of NADP^+^ is Ser-634. Unfortunately, as no full-length structure has been determined for the BM3 CPR domain, the Ser-634 residue is not represented in the CPR model and so the HDX data for Ser-634 and its associated loop region cannot be mapped accurately (38). The deuterium uptake for this residue showed very little change across different state comparisons. However, the deuterium uptake for Ser-634 from the raw data is very high (32–36%), suggesting this amino acid has high solvent accessibility in any conformational state.

In conclusion, we have probed the surface of the biotechnologically important P450-CPR fusion protein P450 BM3. We have identified many areas of interest with respect to sites of protein dimerization and *e.g.* conformational rearrangements associated with ligand binding in this multidomain dimeric enzyme. Important findings include HDX-MS studies that reveal the flexible nature of the CPR domain and its greater mobility in the presence of the heme domain in full-length P450 BM3 enzyme. Structural reorganization was also observed on the binding of NPG to the intact BM3 enzyme, resulting in deshielding in the heme domain. Furthermore, we have identified several additional unfolding intermediates for the full-length protein and heme domain during CIU experiments. In addition, modeling CCS values from X-ray crystallographic structures to our experimental values leads us to believe the CPR domain adopts a more compact structure than the CPR model would suggest. Our findings thus provide important new insights into the structural and conformational properties of this highly dynamic, industrially important protein.

## Experimental procedures

### P450 BM3 expression and purification

WT P450 BM3 and its heme domain were expressed using pET14b (heme domain) and pET15b (full-length P450 BM3 protein) vectors, as described in our previous studies (4). The plasmids were transformed into the *Escherichia coli* BL21 (DE3) strain for gene expression. The BM3 heme domain and the intact P450 BM3 enzyme were expressed in TB medium (Formedium, Hunstanton, UK) with cell growth for 24 h at 37 °C. Cells were harvested by centrifugation (6000 × *g*, 20 min, 4 °C). The cell pellets were resuspended in ice-cold buffer A (50 mm potassium phosphate (potassium P_i_) containing 350 mm KCl and 10% glycerol, pH 8) containing protease inhibitors (1 tablet per 100 ml, EDTA-free cOmplete^TM^ tablets, Roche), and DNase (10 μg ml^−1^) (Merck). Cells were lysed by sonication on ice using a Bandelin Sonopuls instrument at 37% amplitude with 12 pulses for 40 s, and with 60-s breaks between pulses. The cell extract was clarified by centrifugation (4600 × *g*, 60 min, 4 °C). 30% w/v ammonium sulfate was added to the clarified extract and contaminant proteins were removed by incubation for 1 h at 4 °C using gentle agitation. Precipitated material was removed using centrifugation (4600 × *g*, 15 min, 4 °C).

For the full-length P450 BM3 and its heme domain, the proteins were purified using affinity chromatography with a nickel-IDA column, followed by a further chromatography step using hydroxyapatite. The clarified cell extracts in both cases were incubated with nickel-IDA resin overnight at 4 °C in buffer A. The full-length BM3 and heme domain–bound resins were then applied to columns and a stepwise gradient of 10 mm (300 ml), 20 mm (200 ml), and 200 mm (60 ml) imidazole was applied to elute the proteins. The eluted proteins were dialyzed into 25 mm potassium P_i_, pH 6.5 (buffer B) and applied to separate 28-ml columns containing CHT hydroxyapatite type 1 resin (Bio-Rad). A linear gradient of 25–300 mm potassium P_i_, pH 6.5 (600 ml) was applied in both cases to fractionate full-length BM3 and its heme domain.

The CPR domain was cloned into pET11a and expressed in BL21-Gold (DE3) cells. The BM3 CPR domain was expressed in 2× YT medium with cell growth at 37 °C. CPR gene expression was induced when the *A*_600_ reached 0.8 by addition of 500 μm isopropyl 1-thio-β-d-galactopyranoside (Melford Laboratories Ltd., Ipswich, UK). Thereafter, the growth temperature was lowered to 30 °C and cell culture was continued for 24 h. Cells were harvested and resuspended in 50 mm Tris containing 1 mm EDTA, pH 7.2 (buffer C). Subsequently, the cells were ruptured by sonication and the CPR domain partially purified using 30% w/v ammonium sulfate, as described above for the full-length BM3 and heme domain proteins. The CPR protein required dialysis before further purification to remove the ammonium sulfate. The dialysis was completed using buffer C. The CPR was purified using a 150 ml DEAE Sepharose^TM^ fast flow resin (GE Healthcare Life Sciences) with a linear gradient of 0–300 mm KCl in buffer C (1500 ml). The protein was dialyzed into 50 mm potassium P_i_, pH 7.2 (buffer D) for further purification on a Q-Sepharose^TM^ Fast Flow column. The CPR protein was then eluted using a gradient of 0–300 mm KCl in buffer D. Finally, the protein was purified using a hydroxyapatite column as described above for the full-length P450 BM3 and heme domain proteins.

Before analysis of the purified proteins, the samples were desalted using a gel filtration column (HiLoad® GF S200 16/600 Superdex® 200 pg, GE Healthcare Life Sciences). 50 mm ammonium acetate, pH 7 (buffer E) was the buffer chosen because of its volatility for use in native MS experiments.

### Native MS and IM-MS

On the day of analysis, the protein was further desalted by exchange into 100 mm ammonium acetate (buffer E) using Micro Bio-Spin Chromatography columns (Bio-Rad, Micro Bio-Spin 6 Columns), following the manufacturer's instructions. The buffer was exchanged three times for the full-length P450 BM3 enzyme and once for the heme and CPR domains to ensure adequate protein compatibility for native MS. Native MS and IM-MS data were acquired on a Synapt G2S HDMS instrument (Waters, Manchester, UK). NanoESI capillaries were prepared in house from thin-walled borosilicate capillaries (inner diameter 0.9 mm, outer diameter 1.2 mm) (World Precision Instruments, Stevenage, UK) using a Flaming/Brown P-97 micropipette puller (Sutter Instrument Company, Novato, CA). A positive voltage was applied to the solution through a platinum wire (Goodfellow Cambridge Ltd., Huntington, UK) inserted into the capillary. Gentle source conditions were applied to preserve the native-like structure: capillary voltage 1.2–1.5 kV, sampling cone 40 V, source temperature 40 °C. Trap collision energy was 4 V and transfer collision energy was set to 0 V. IM-MS experiments were acquired using a modified Waters Synapt G2 with a 25.05 cm Radio-Frequency confining linear drift cell filled with ∼2 Torr helium, 298 K. A multifield approach (20 V drift voltage increments) was applied using WREnS and in-house software (ORIGAMI) (39). Capillary voltage was 1.3–1.6 kV, cone 60 V, and source temperature 40 °C. The arrival time distributions were converted to CCS. Measurements were performed in helium as well as in nitrogen as the carrier gas. External calibration of the spectra was achieved using solutions of cesium iodide (2 mg/ml in 50:50 water:isopropanol). Data were acquired and processed with MassLynx software (Waters).

### CIU

Experiments were performed on a Waters Synapt G2S instrument using NanoESI and trap-activated ion mobility; capillary voltage was 1.2–1.5 kV, cone 40 V, and source temperature 40 °C. The helium cell and the IMS gas flows were 180 and 90 ml/min, respectively; the IMS wave velocity was 400 m/s, and the IMS wave height was 35 V. Nitrogen was the carrier gas. The most intense charge state for each protein species was mass selected using the quadrupole prior to the trap region. Activation was induced by elevating the trap collision energy. ORIGAMI was used to automatically acquire data for collision energies from 4 to 200 V in 2-V increments, as well as for data processing (39).

### Calculating CCS values from X-ray crystallographic structures

Structures were first energy minimized in the gas phase (with an infinite nonbonded cut-off) using AmberTools14 (40) and the Amber14 force field (41). CCS values were then calculated using the trajectory method (42), as implemented in the IMoS suite (42–44). The CCS values were calculated for the crystal structure 1BU7 for the BM3 heme domain. For the CPR domain, a model was created using PDB 1BVY (the BM3 FMN domain) and PDB 4DQK (the BM3 FAD domain) aligned to PDB 1AMO (the rat CPR structure), as no full-length BM3 CPR structure has been solved to date.

### HDX-MS

The same protein stocks for native MS were also used for HDX-MS. The protein was incubated with appropriate ligands for a few hours on ice. The ligands used were 0.5 mm *N*-palmitoylglycine (NPG) and/or 1 mm NADPH (NADP^+^), which bind in the P450 active site and to the FAD domain, close to the FAD cofactor, respectively. NPG and NADP^+^ were purchased from the Cayman Chemical Company (Ann Arbor, Michigan) and Bio Basic Canada Inc. (Markham, Canada). Samples were frozen at −80 °C until required.

The HDX-MS set-up was comprised of a Waters nanoACQUITY UPLC system with ESI-MS detection coupled to a LEAP Technologies dual-armed robot for sample preparation, incubation, and inlet injection. A Waters Synapt G2S mass spectrometer was operated in positive ion/resolution mode, with data acquired over the *m*/*z* range 290–2500. 30 μm protein solutions were diluted 20-fold into 10 mm potassium P_i_ in either H_2_O or D_2_O, pH/pD 7, and the mixture incubated at 20 °C for 0 min (in H_2_O) or for 1, 10, 30, or 180 min (in D_2_O), before the quench step. HDX quenching was achieved by mixing the reaction solution 1:1 with cooled 100 mm potassium P_i_ (pH 2.5, 0 °C). Approximately 37.5 pmol were then injected into the HDX module (0 °C) and washed over a pepsin column (Waters Enzymate BEH Pepsin, 2.1 × 30 mm) with 0.1% HCOOH in H_2_O, pH 2.5, at 200 μl min^−1^. Resulting peptides were trapped on a VanGuard C18 trap column. Peptide separation was achieved on a C18 column (Waters ACQUITY UPLC BEH C18 1.7 μm, 1.0 × 10 mm) at 40 μl/min flow over 16 min with the following gradient: 0 min, 5% B; 7 min, 35% B; 8 min, 85% B; 11 min, 5% B; 12 min, 95% B; 13 min, 5% B; 14 min, 95% B; 15 min, 5% B (mobile phases: A, water + 0.1% formic acid; and B, acetonitrile + 0.1% formic acid). The mass spectrometer was operated in TOF mode (no IM), with MS^e^ data acquisition (trap collision energy ramp 14–35 V). LeuEnk peptide was used as Lock Spray. Data were acquired using Waters MassLynx software v4.1, with the LEAP robot controlled by HDx Director 1.0.3.9. Data processing and analysis were carried out with Waters ProteinLynx Global Server 3.0.1 and Waters DynamX 3.0 software, respectively. Python scripts of the deuterium uptake were generated using the data collected at 1 h. Python scripts were mapped onto structures in PyMOL (The PyMOL Molecular Graphics System, Version 1.7.4.5 Schrödinger, LLC). High coverage was generated for each state; full-length BM3 *versus* heme domain (91.5%), full-length BM3 *versus* CPR domain (89.8%), full-length BM3/no ligands *versus* full-length BM3/plus ligands (99.1%).

## Data availability

All the data presented are available in this manuscript.

## Author contributions

L. N. J., P. E. B., M. W. V., and A. W. M. conceptualization; L. N. J., K. J. P., L. O. J., and H. M. G. data curation; L. N. J., K. J. P., L. O. J., and H. M. G. validation; L. N. J., K. J. P., H. M. G., and M. W. V. investigation; L. N. J., K. J. P., L. O. J., H. M. G., P. E. B., M. W. V., and A. W. M. methodology; L. N. J., H. M. G., and A. W. M. writing-original draft; L. N. J., K. J. P., H. M. G., P. E. B., M. W. V., and A. W. M. writing-review and editing; K. J. P., and P. E. B. resources; L. O. J. software; P. E. B., M. W. V., and A. W. M. supervision; A. W. M. funding acquisition; A. W. M. project administration.

## Supplementary Material

Supporting Information

## Footnotes

2 The abbreviations used are:

BM3: cytochrome P450 BM3
CPR: cytochrome P450 reductase
IM-MS: ion mobility MS
CIU: collision-induced unfolding
HDX-MS: hydrogen-deuterium exchange MS
NPG: *N-*palmitoylglycine
DSC: differential scanning calorimetry
CCS: collision cross section.
