# Supplementary material for: Characterization of the structure and interactions of P450 BM3 using hybrid mass spectrometry approaches
Source: J Biol Chem. 2020 Apr 17;295(22):7595–607. doi: 10.1074/jbc.RA119.011630 (PMC7261786; doi:10.1074/jbc.RA119.011630)
Supplement: Supporting Information [file supp_295_22_7595__index.html]

Characterization of the structure and interactions of P450 BM3 using hybrid mass spectrometry approaches — Molecular interactions in cytochrome P450 BM3 — Supporting Information 

# Characterization of the structure and interactions of P450 BM3 using hybrid mass spectrometry approaches

## Supporting Information

- Supporting Information (to be published online) - supporting information to be published along with the main paper
