# Supplementary material for: Characterization of the structure and interactions of P450 BM3 using hybrid mass spectrometry approaches
Source: J Biol Chem. 2020 Apr 17;295(22):7595–607. doi: 10.1074/jbc.RA119.011630 (PMC7261786; doi:10.1074/jbc.RA119.011630)
Supplement: Supporting Information [file supp_RA119.011630_156712_2_supp_503018_q8xky2.pdf]

SUPPORTING INFORMATION

Characterization of the structure and interactions of P450 BM3 using hybrid mass spectrometry approaches

Laura N. Jeffreys[1], Kamila J. Pacholarz[2], Linus O. Johannissen[1], Hazel M. Girvan[1], Perdita E. Barran[1,2], Michael W. Voice[3] and Andrew W. Munro[1*]

[1]The Manchester Institute of Biotechnology, School of Natural Sciences, Department of Chemistry, The University of Manchester, 131 Princess Street, Manchester M1 7DN, UK; and the [1]Manchester Synthetic Biology Research Centre for Fine and Speciality Chemicals (SYNBIOCHEM), The University of Manchester, 131 Princess Street, Manchester M1 7DN, UK; [2]Michael Barber Centre for Collaborative Mass Spectrometry, Manchester Institute of Biotechnology, University of Manchester 131 Princess Street, Manchester M1 7DN, UK; [3]Cypex Ltd., 6 Tom McDonald Avenue, Dundee, DD2 1NH, UK.

Table of contents

Figure S1 - Native MS of full-length P450 BM3 and its component domains in ligand-free and ligand-bound states

Table S1 - Experimentally and theoretically derived collision cross-section (CCS) values for full-length P450 BM3 and its domains

Figure S2 - Relative deuterium uptake plots for ligand-free and ligand-bound full-length P450 BM3 dimeric protein

Figure S3 - Coverage maps for the ligand-free P450 BM3 heme and CPR domains

Figure S4 - Coverage maps for the ligand-bound, full-length P450 BM3 dimeric protein

Figure S5 - The P450 BM3 residues showing the highest degree of change when comparing full-length P450 BM3 with its isolated domains

Figure S6 - The P450 BM3 residues showing the highest degree of change when comparing ligand-free and ligand-bound full-length P450 BM3

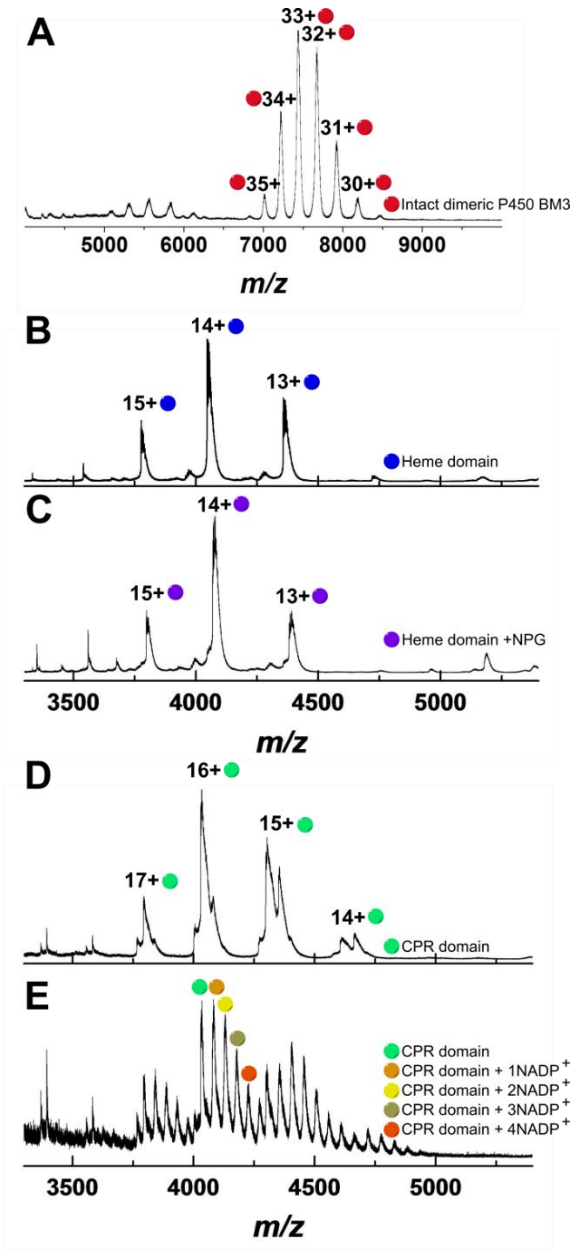

Figure S1. Native MS of full-length P450 BM3 and its component domains in ligand-free and ligand-bound states. Panel A: The native MS spectrum of full-length P450 BM3 shows multiple charge states as well as the presence of dimeric (major component) and monomeric (minor component) protein. **Panel B**: The native MS spectrum of the heme domain protein shows only three prevalent charge states. **Panel C**: The MS spectrum of the heme domain does not change significantly upon association with the tight binding BM3 substrate NPG. **Panel D**: The MS spectrum of the CPR domain shows a number of different charge states. **Panel E**: The MS spectrum of the CPR domain changes substantially on the binding of $NADP^+$ to the CPR. Multiple species are observed, corresponding to 1, 2, 3 and 4 molecules of $NADP^+$ binding to the CPR domain.

Table S1. Experimentally and theoretically derived collision cross-section (CCS) values for full-length P450 BM3 and its domains. CCS values were determined for the full-length, dimeric P450 BM3 protein and its component heme and reductase (CPR) domains using IM-MS with helium as the carrier gas. In addition, CCS values were determined for ligand-bound domains. Theoretical CCS values were calculated using crystallographic structures (1BU7, 4DQK and 1BVY) using the IMoS suite with helium as the carrier gas. These calculations gave CCS values of 39.1 nm^2 for the heme domain (13% difference to the experimental data) and 46.1 nm^2 for the reductase domain model (23% difference to the experimental data).

**Experimental**			**Theoretical**	**Expected mass**	**Measured mass**
**Protein**	**CCS (nm^2)**	**STDEV**	**ImoS (nm^2)**	**Da**	**Da**
Full-length protein	99.66	1.21	-	239889.20	244915.91 ± 76.08
Heme domain	34.56	0.17	39.1	56147.08	56637.51 ± 0.71
Heme domain + NPG	34.71	0.24	-	56406.58	56952.95 ± 1.56
CPR domain	37.53	1.08	46.1	64145.98	64536.33 ± 30.81
CPR domain + 1 $NADP^+$	37.05	0.38	-	64889.38	65229.05 ± 11.67
CPR domain + 2 $NADP^+$	37.39	0.15	-	65632.78	66054.80 ± 2.79
CPR domain + 3 $NADP^+$	38.03	0.48	-	66376.18	66809.45 ± 14.95
CPR domain + 4 $NADP^+$	38.31	0.43	-	67119.58	67563.77 ± 20.17

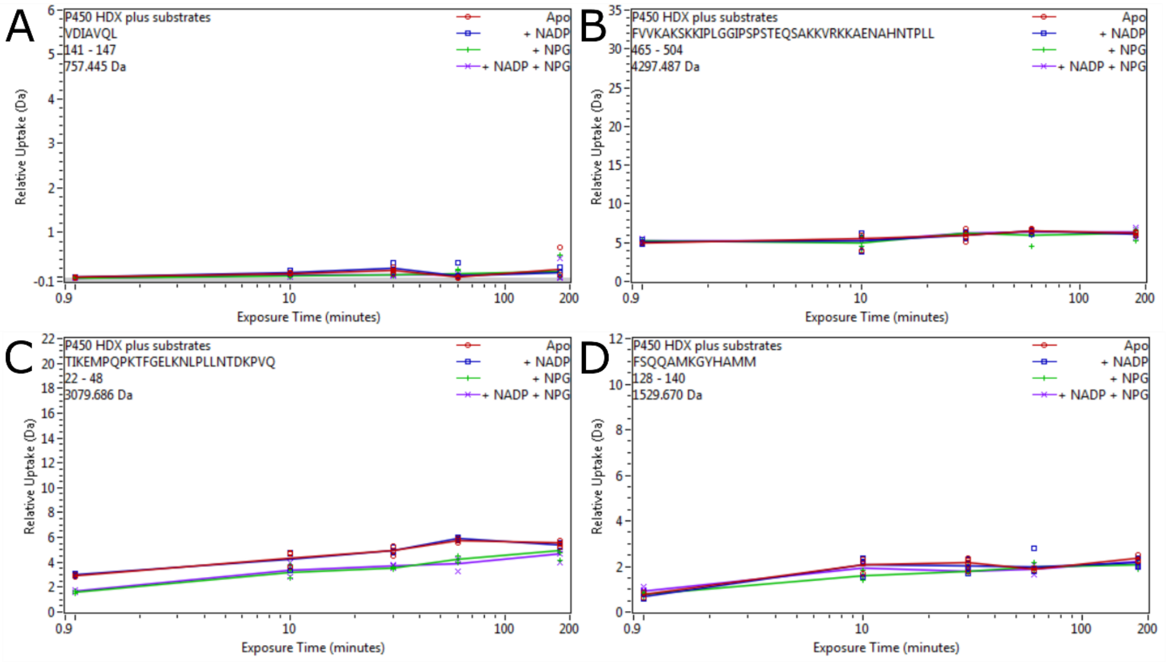

Figure S2. Relative deuterium uptake plots for ligand-free and ligand-bound full-length P450 BM3 dimeric protein. Using a LEAP Technologies dual-armed robot, accurate deuterium incubations could be accomplished for HDX-MS analysis. Observing the deuterium uptake from 1 minute to 180 minutes for each peptide fragment allows determination of the solvent accessibility of the protein. Four states are compared above; ligand-free (red), $NADP^+$-bound (blue), NPG-bound (green) and $NADP^+$/NPG-bound (purple). Plots were generated using Waters DynamX software. In Panel A a small segment of the protein exhibits very poor deuterium uptake over time. In Panel B a longer segment of the protein exhibits poor deuterium uptake over time. However, this segment begins with higher deuterium levels than Panel A. In Panels C and D deuterium uptake increases over time. However, in Panel C there is an observable difference between different ligand-bound states.

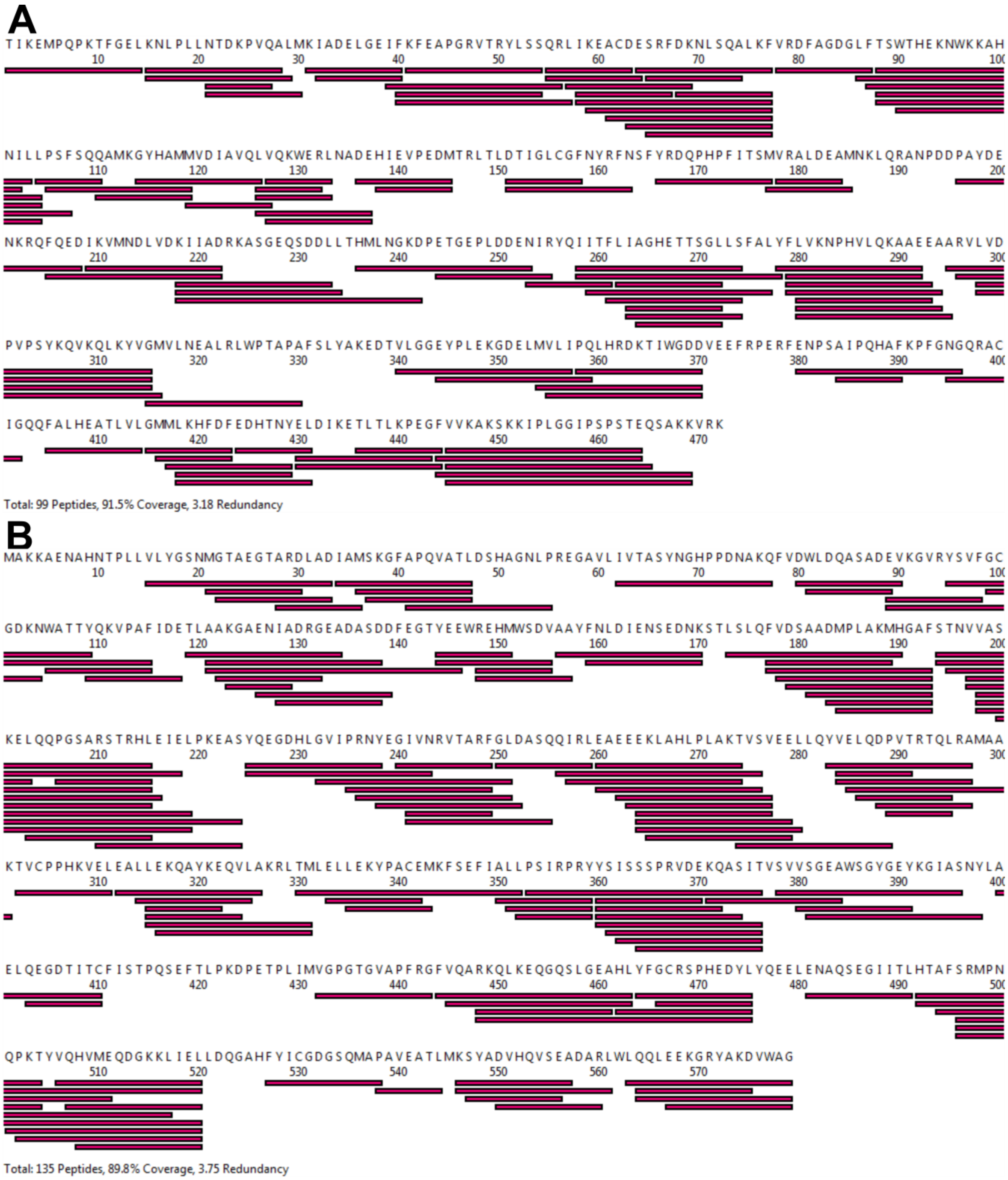

Figure S3. The coverage maps for the ligand-free P450 BM3 heme and CPR domains. Panel A: The heme domain HDX-MS data show 91.5% coverage with 3.18 redundancy. **Panel B:** The CPR domain data show 89.8% coverage with 3.75 redundancy.

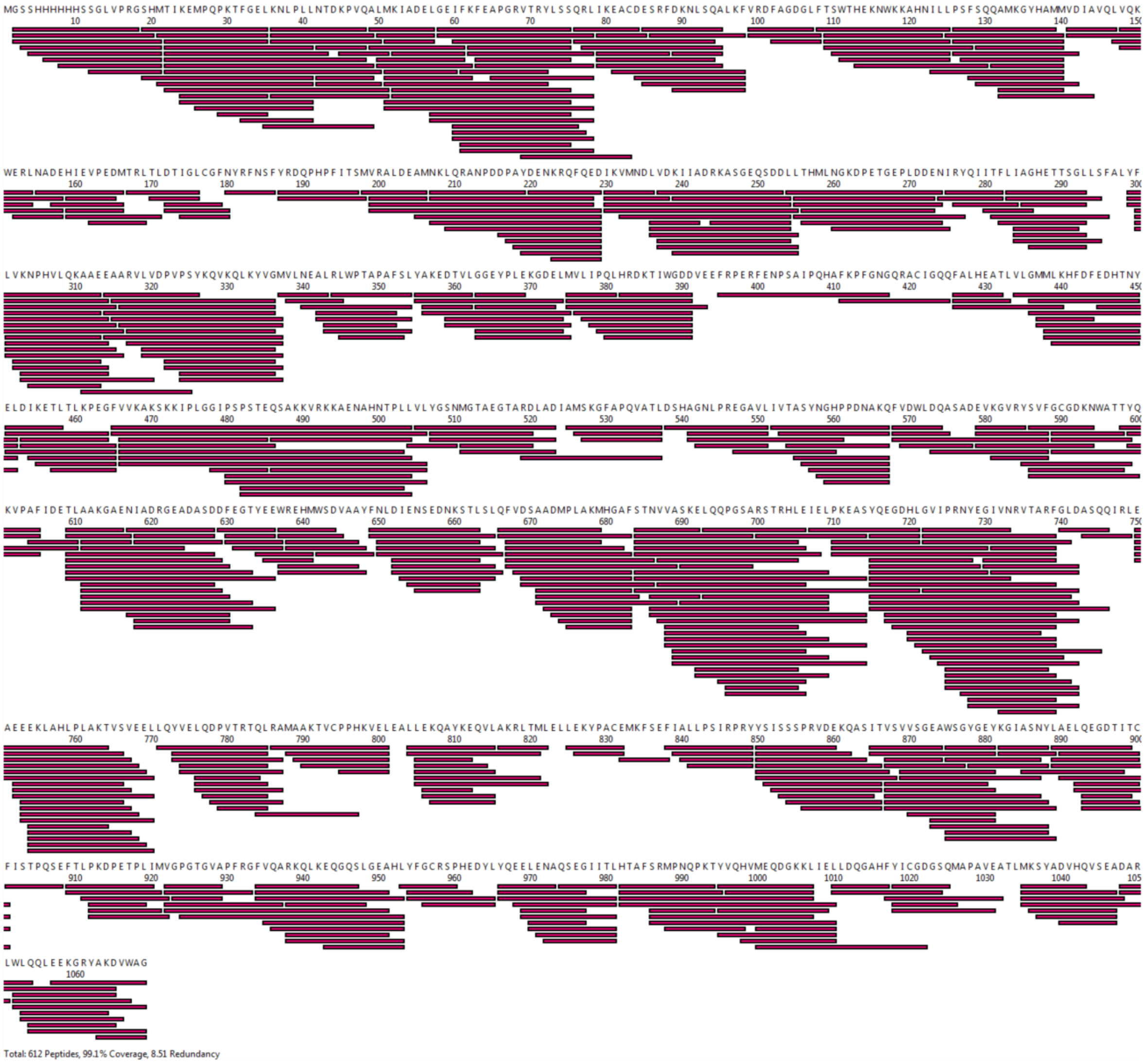

Figure S4. The coverage maps for the ligand-bound, full-length P450 BM3 dimeric protein. All four states of the protein (ligand-free, NADP$^+$-bound, NPG-bound and NADP$^+$/NPG-bound) show 99.1% coverage and 8.51 redundancy.

```
MGSSHHHHHH SSGLVPRGSH MTIKEMPQPK TFGELKNLPL LNTDKPVQAL MKIADELGEI
FKFEAPGRVT RYLSSQRLIK EACDESRFDK NLSQALKFVR DFAGDGLFTS WTHEKNWKKA
HNILLPSFSQ QAMKGYHAMM VDIAVQLVQK WERLNADEHI EVPEDMTRLT LDTIGLCGFN
YRFNSFYRDQ PHPFITSMVR ALDEAMNKLQ RANPDDPAYD ENKRQFQEDI KVMNDLVDKI
IADRKASGEQ SDDLLTHMLN GKDPETGEPL DDENIRYQII TFLIAGHETT SGLLSFALYF
LVKNPHVLQK AAEEAARVLV DPVPSYKQVK QLKYVGMVLN EALRLWPTAP AFSLYAKEDT
VLGGEYPLEK GDELMVLIPQ LHRDKTIWGD DVEEFRPERF ENPSAIPQHA FKPFGNGQRA
CIGQQFALHE ATLVLGMMLK HFDFEDHTNY ELDIKETLTL KPEGFVVKAK SKKIPLGGIP
SPSTEQSAKK VRKKAENAHN TPLLVLYGSN MGTAEGTARD LADIAMSKGF APQVATLDSH
AGNLPREGAV LIVTASYNGH PPDNAKQFVD WLDQASADEV KGVRYSVFGC GDKNWATTYQ
KVPAFIDETL AAKGAENIAD RGEADASDDF EGTYEEWREH MWSDVAAYFN LDIENSEDNK
STLSLQFVDS AADMPLAKMH GAFSTNVVAS KELQQPGSAR STRHLEIELP KEASYQEGDH
LGVIPRNYEG IVNRVTARFG LDASQQIRLE AEEEKLAHLP LAKTVSVEEL LQYVELQDPV
TRTQLRAMAA KTVCPPHKVE LEALLEKQAY KEQVLAKRLT MLELLEKYPA CEMKFSEFIA
LLPSIRPRYY SISSSPRVDE KQASITVSVV SGEAWSGYGE YKGIASNYLA ELQEGDTITC
FISTPQSEFT LPKDPETPLI MVGPGTGVAP FRGFVQARKQ LKEQGQSLGE AHLYFGCRSP
HEDYLYQEEL ENAQSEGIIT LHTAFSRMPN QPKTYVQHVM EQDGKKLIEL LDQGAHFYIC
GDGSQMAPAV EATLMKSYAD VHQVSEADAR LWLQQLEEKG RYAKDVWAG*
```

Figure S5. The P450 BM3 residues showing the highest degree of change when comparing full-length P450 BM3 with its isolated domains. The residues are coloured red or cyan to indicate areas of the greatest deshielding or shielding respectively (±5% deuterium uptake), when subtracting the isolated domains from the full-length P450 BM3 protein. These residues correspond to conformational changes causing alterations in solvent accessibility. In particular, the residues highlighted in cyan correspond to the sites hypothesized to be involved with dimerization. A His-tag was used to facilitate protein purification and is shown in green. Residues with no coverage are not coloured.

A

```
MGSSHHHHHH SSGLVPRGSH MTIKEMPQPK TFGELKNLPL LNTDKPVQAL MKIADELGEI
FKFEAPGRVT RYLSSQRLIK EACDESRFDK NLSQALKFVR DFAGDGLFTS WTHEKNWKKA
HNILLPSFSQ QAMKGYHAMM VDIAVQLVQK WERLNADEHI EVPEDMTRLT LDTIGLCGFN
YRFNSFYRDQ PHPFITSMVR ALDEAMNKLQ RANPDDPAYD ENKRQFQEDI KVMNDLVDKI
IADRKASGEQ SDDLLTHMLN GKDPETGEPL DDENIRYQII TFLIAGHETT SGLLSFALYF
LVKNPHVLQK AAEEAARVLV DPVPSYKQVK QLKYVGMVLN EALRLWPTAP AFSLYAKEDT
VLGGEYPLEK GDELMVLIPQ LHRDKTIWGD DVEEFRPERF ENPSAIPQHA FKPFGNGQRA
CIGQQFALHE ATLVLGMMLK HFDFEDHTNY ELDIKETLTL KPEGFVVKAK SKKIPLGGIP
SPSTEQSAKK VRKKAENAHN TPLLVLYGSN MGTAEGTARD LADIAMSKGF APQVATLDSH
AGNLPREGAV LIVTASYNGH PPDNAKQFVD WLDQASADEV KGVRYSVFGC GDKNWATTYQ
KVPAFIDETL AAKGAENIAD RGEADASDDF EGTYEEWREH MWSDVAAYFN LDIENSEDNK
STLSLQFVDS AADMPLAKMH GAFSTNVVAS KELQQPGSAR STRHLEIELP KEASYQEGDH
LGVIPRNYEG IVNRVTARFG LDASQQIRLE AEEEKLAHLP LAKTVSVEEL LQYVELQDPV
TRTQLRAMAA KTVCPPHKVE LEALLEKQAY KEQVLAKRLT MLELLEKYPA CEMKFSEFIA
LLPSIRPRYY SISSSPRVDE KQASITVSVV SGEAWSGYGE YKGIASNYLA ELQEGDTITC
FISTPQSEFT LPKDPETPLI MVGPGTGVAP FRGFVQARKQ LKEQGQSLGE AHLYFGCRSP
HEDYLYQEEL ENAQSEGIIT LHTAFSRMPN QPKTYVQHVM EQDGKKLIEL LDQGAHFYIC
GDGSQMAPAV EATLMKSYAD VHQVSEADAR LWLQQLEEKG RYAKDVWAG*
```

B

```
MGSSHHHHHH SSGLVPRGSH MTIKEMPQPK TFGELKNLPL LNTDKPVQAL MKIADELGEI
FKFEAPGRVT RYLSSQRLIK EACDESRFDK NLSQALKFVR DFAGDGLFTS WTHEKNWKKA
HNILLPSFSQ QAMKGYHAMM VDIAVQLVQK WERLNADEHI EVPEDMTRLT LDTIGLCGFN
YRFNSFYRDQ PHPFITSMVR ALDEAMNKLQ RANPDDPAYD ENKRQFQEDI KVMNDLVDKI
IADRKASGEQ SDDLLTHMLN GKDPETGEPL DDENIRYQII TFLIAGHETT SGLLSFALYF
LVKNPHVLQK AAEEAARVLV DPVPSYKQVK QLKYVGMVLN EALRLWPTAP AFSLYAKEDT
VLGGEYPLEK GDELMVLIPQ LHRDKTIWGD DVEEFRPERF ENPSAIPQHA FKPFGNGQRA
CIGQQFALHE ATLVLGMMLK HFDFEDHTNY ELDIKETLTL KPEGFVVKAK SKKIPLGGIP
SPSTEQSAKK VRKKAENAHN TPLLVLYGSN MGTAEGTARD LADIAMSKGF APQVATLDSH
AGNLPREGAV LIVTASYNGH PPDNAKQFVD WLDQASADEV KGVRYSVFGC GDKNWATTYQ
KVPAFIDETL AAKGAENIAD RGEADASDDF EGTYEEWREH MWSDVAAYFN LDIENSEDNK
STLSLQFVDS AADMPLAKMH GAFSTNVVAS KELQQPGSAR STRHLEIELP KEASYQEGDH
LGVIPRNYEG IVNRVTARFG LDASQQIRLE AEEEKLAHLP LAKTVSVEEL LQYVELQDPV
TRTQLRAMAA KTVCPPHKVE LEALLEKQAY KEQVLAKRLT MLELLEKYPA CEMKFSEFIA
LLPSIRPRYY SISSSPRVDE KQASITVSVV SGEAWSGYGE YKGIASNYLA ELQEGDTITC
FISTPQSEFT LPKDPETPLI MVGPGTGVAP FRGFVQARKQ LKEQGQSLGE AHLYFGCRSP
HEDYLYQEEL ENAQSEGIIT LHTAFSRMPN QPKTYVQHVM EQDGKKLIEL LDQGAHFYIC
GDGSQMAPAV EATLMKSYAD VHQVSEADAR LWLQQLEEKG RYAKDVWAG*
```

C

```
MGSSHHHHHH SSGLVPRGSH MTIKEMPQPK TFGELKNLPL LNTDKPVQAL MKIADELGEI
FKFEAPGRVT RYLSSQRLIK EACDESRFDK NLSQALKFVR DFAGDGLFTS WTHEKNWKKA
HNILLPSFSQ QAMKGYHAMM VDIAVQLVQK WERLNADEHI EVPEDMTRLT LDTIGLCGFN
YRFNSFYRDQ PHPFITSMVR ALDEAMNKLQ RANPDDPAYD ENKRQFQEDI KVMNDLVDKI
IADRKASGEQ SDDLLTHMLN GKDPETGEPL DDENIRYQII TFLIAGHETT SGLLSFALYF
LVKNPHVLQK AAEEAARVLV DPVPSYKQVK QLKYVGMVLN EALRLWPTAP AFSLYAKEDT
VLGGEYPLEK GDELMVLIPQ LHRDKTIWGD DVEEFRPERF ENPSAIPQHA FKPFGNGQRA
CIGQQFALHE ATLVLGMMLK HFDFEDHTNY ELDIKETLTL KPEGFVVKAK SKKIPLGGIP
SPSTEQSAKK VRKKAENAHN TPLLVLYGSN MGTAEGTARD LADIAMSKGF APQVATLDSH
AGNLPREGAV LIVTASYNGH PPDNAKQFVD WLDQASADEV KGVRYSVFGC GDKNWATTYQ
KVPAFIDETL AAKGAENIAD RGEADASDDF EGTYEEWREH MWSDVAAYFN LDIENSEDNK
STLSLQFVDS AADMPLAKMH GAFSTNVVAS KELQQPGSAR STRHLEIELP KEASYQEGDH
LGVIPRNYEG IVNRVTARFG LDASQQIRLE AEEEKLAHLP LAKTVSVEEL LQYVELQDPV
TRTQLRAMAA KTVCPPHKVE LEALLEKQAY KEQVLAKRLT MLELLEKYPA CEMKFSEFIA
```

LLPSIRPRYY SISSSPRVDE KQASITVSVV SGEAWSGYGE YKGIASNYLA ELQEGDTITC
FISTPQSEFT LPKDPETPLI MVGPGTGVAP FRGFVQARKQ LKEQGQSLGE AHLYFGCRSP
HEDYLYQEEL ENAQSEGIIT LHTAFSRMPN QPKTYVQHVM EQDGKKLIEL LDQGAHFYIC
GDGSQMAPAV EATLMKSYAD VHQVSEADAR LWLQQLEEKG RYAKDVWAG*

Figure S6. The P450 BM3 residues showing the highest degree of change when comparing ligand-free and ligand-bound full-length P450 BM3. The residues are coloured red or cyan to indicate areas of the greatest deshielding or shielding respectively (±5% deuterium uptake), when subtracting ligand-free values from NADP$^+$-bound (A), NPG-bound (B) and NADP$^+$/NPG-bound protein (C). These residues correspond to conformational changes causing alterations in solvent accessibility. In particular, the residues highlighted in cyan correspond to the sites hypothesized to be involved with dimerization. The His-tag used to facilitate protein purification is shown in green. Residues with no coverage are not coloured.
